# Adaptive Radiation Therapy in the Treatment of Lung Cancer: An Overview of the Current State of the Field

**DOI:** 10.3389/fonc.2021.770382

**Published:** 2021-11-29

**Authors:** Huzaifa Piperdi, Daniella Portal, Shane S. Neibart, Ning J. Yue, Salma K. Jabbour, Meral Reyhan

**Affiliations:** ^1^ Department of Radiation Oncology, Rutgers Cancer Institute of New Jersey, New Brunswick, NJ, United States; ^2^ Rutgers Robert Wood Johnson Medical School, Rutgers, The State of New Jersey University, Piscataway, NJ, United States

**Keywords:** adaptive radiation therapy (ART), adaptive planning, non-small cell lung cancer, small cell lung cancer (SCLC), lung cancer

## Abstract

Lung cancer treatment is constantly evolving due to technological advances in the delivery of radiation therapy. Adaptive radiation therapy (ART) allows for modification of a treatment plan with the goal of improving the dose distribution to the patient due to anatomic or physiologic deviations from the initial simulation. The implementation of ART for lung cancer is widely varied with limited consensus on who to adapt, when to adapt, how to adapt, and what the actual benefits of adaptation are. ART for lung cancer presents significant challenges due to the nature of the moving target, tumor shrinkage, and complex dose accumulation because of plan adaptation. This article presents an overview of the current state of the field in ART for lung cancer, specifically, probing topics of: patient selection for the greatest benefit from adaptation, models which predict who and when to adapt plans, best timing for plan adaptation, optimized workflows for implementing ART including alternatives to re-simulation, the best radiation techniques for ART including magnetic resonance guided treatment, algorithms and quality assurance, and challenges and techniques for dose reconstruction. To date, the clinical workflow burden of ART is one of the major reasons limiting its widespread acceptance. However, the growing body of evidence demonstrates overwhelming support for reduced toxicity while improving tumor dose coverage by adapting plans mid-treatment, but this is offset by the limited knowledge about tumor control. Progress made in predictive modeling of on-treatment tumor shrinkage and toxicity, optimizing the timing of adaptation of the plan during the course of treatment, creating optimal workflows to minimize staffing burden, and utilizing deformable image registration represent ways the field is moving toward a more uniform implementation of ART.

## Introduction

Lung cancer treatment is constantly evolving. Cytotoxic chemotherapeutic regimens, once the cornerstone of management of advanced disease, have been largely augmented by targeted therapy and immune checkpoint inhibitors. While these systemic options have disrupted the field, technological advances in the delivery of radiation therapy have also dramatically changed over recent decades, now incorporating 4D computed tomography (4DCT), highly conformal treatment techniques including intensity modulated radiation therapy (IMRT), and adaptive radiation therapy (ART).

ART allows for modification of a treatment plan with the goal of improving the dose distribution to the patient due to anatomic or physiologic deviations from the initial simulation (1). Conventionally, lung cancer radiation treatment planning begins with 4DCT simulation, generating a snapshot of the tumor size, shape, and position relative to normal tissue, which is used for the creation of an internal gross tumor volume (iGTV) or internal target volume (ITV). While the technologies to treat these tumors allow for highly conformal dose distributions, the complex geometric uncertainties involved in lung cancer treatment planning require large safety margins to create the planning target volume (PTV) (2), which may hamper dose escalation. Additionally, planning weeks ahead of radiation treatment initiation based on images at one point of time potentially risks poor tumor coverage and more severe off-target effects. ART addresses these weaknesses by enabling periodic changes to the treatment plan.

**Figure 1:**
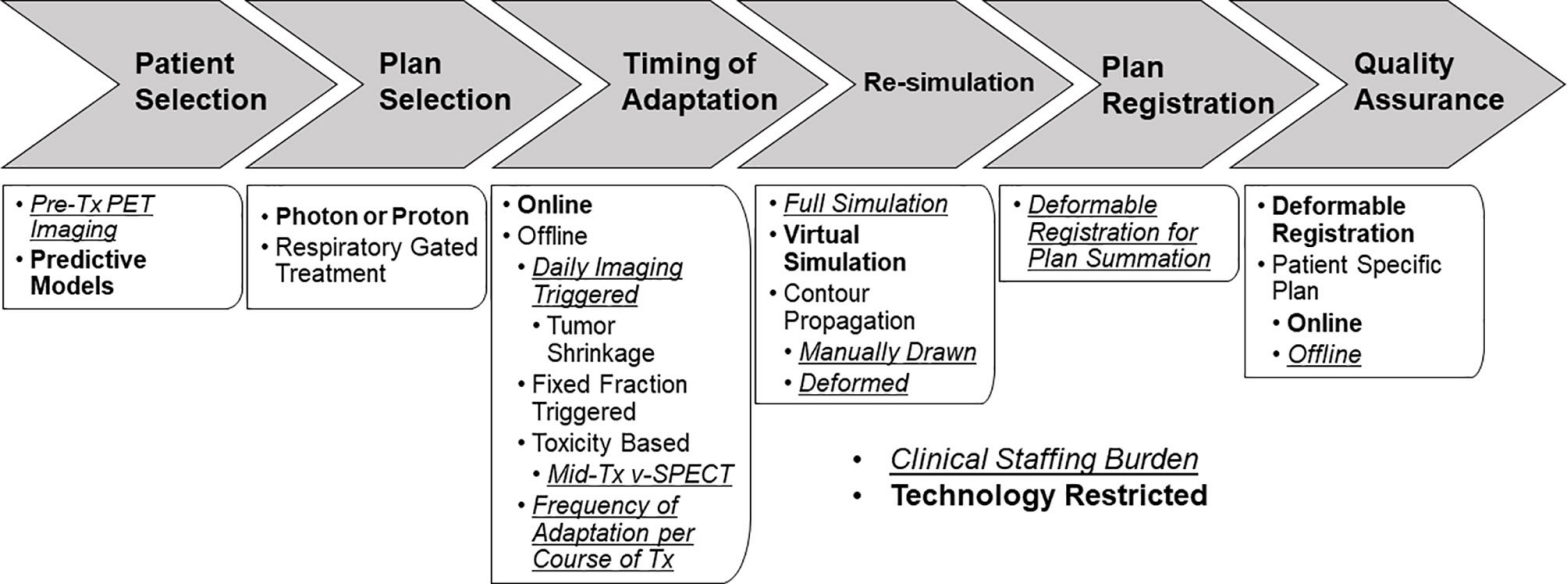
ART workflow for lung cancer patients highlighting diverging workflow, decision points, technology restrictions (bold text), and additional staffing burden (italic/underlined text).

Modern ART can be classified into three categories: offline, online, and real-time adaptation. Offline adaptation refers to the process of updating the patient’s treatment plan after the delivery of a single or multiple fractions of treatment, often involving re-simulation, re-contouring, and re-planning in the same manner the initial treatment plan was created. Online adaptation is a rapidly developing field that utilizes plan adaptations immediately before the delivery of the fraction, often involving re-contouring and re-planning on an IGRT derived imaging dataset. Real-time adaptation automatically adapts the treatment plan during the fraction of treatment, based on real-time imaging to gate the treatment beam or track the target using the multi-leaf collimator (MLC) (3). All forms of ART place a substantial personnel burden on the department: the physician may need to create new contours, review/approve contours, and review/approve the adapted plan; the dosimetrist/physicist may need to create a new plan; and the physicist may need to perform quality assurance (QA) on the adapted plan or add additional QA procedures to the existing QA program due to implementation of new technologies to facilitate ART. However, the staffing burden may be outweighed by the potential benefits of better dosimetric coverage of the tumor and sparing of dose to the organs at risk (OARs). The implementation of ART for lung cancer is widely varied with limited consensus on which patients to adapt, the timing of adaptation, techniques for adaptation, and the actual benefits of adaptation (**Figure 1**). ART for lung cancer presents significant challenges due to the nature of the moving target, tumor shrinkage, and complex dose accumulation because of plan adaptation. Existing review papers on adaptive radiation for lung cancer (4, 5) examined results from larger prospective clinical trials in order to describe the methods and benefits of ART. This article aims to provide a more extensive review of the state of the field by conducting a literature search. This review article presents an overview of the current state of the field in ART for lung cancer, specifically probing topics of: patient selection for the greatest benefit from adaptation, models which predict who/when to adapt plans, best timing for plan adaptation, optimized workflows for implementing ART including alternatives to re-simulation, the best radiation techniques for ART including magnetic resonance (MR) guided treatment, algorithms and QA, and challenges and techniques for dose reconstruction.

## Methods

A literature was conducted in PubMed from 1/2007-12/2020 using terms of: [(“lung cancer”) OR (“NSCLC”) OR (“SCLC”)] AND [(“adaptive radiotherapy”) OR (“adaptive planning”)]. The search produced 268 results, 100 of which were determined to be relevant to the review. Studies were excluded due to lack of mention of or discussion of ART, for diseases other than lung cancer, and presenting inconclusive findings or providing limited application such as a case study. Of the 100 relevant articles, 53 studies were deemed impactful for providing significant conclusions to the review. **Table 1** summarizes the studies selected by this search process, describing the sample size, study type, patient population, treatment technique, dose regimen, type of adaptive planning, and timing of adaptation.

**Table 1:** Select study characteristics from studies published from 2007-2020 on ART for lung cancer.

Study	Sample size	Study type	Patient population	Modality	Dose	Adaptive planning	Timing
Koay et al. (6)	N=9	Prospective	Stage IIIA (N=2) and IIIB (N=7) NSCLC	PSPT	74 Gy(RBE), 2 Gy per fraction	4DCT re-simulation	Week 3/4
Jiang et al. (7)	N=97	Retrospective	Stage III NSCLC	IMRT	50-70 Gy, 1.8-2 Gy per fraction	Midtreatment CT-based dose escalation	After 40-50 Gy
Berkovic et al. (8)	N=41	Retrospective	Stage IIIA (N=30), IIIB (N=10), and IV (N=1)	IMRT	62-70 Gy, 2 Gy per fraction	CBCT registration	Fraction 15/20
Appel et al. (9)	N=58	Retrospective	Stage II (3), Stage III (53), Stage IV (2) NSCLC	3DCT (12), IMRT/VMAT (34), Hybrid (12)	50-66 Gy, mean 59.4 Gy	Daily (45), Weekly (13)	Timing of replanning was in the first, second and final third of treatment course in 26%, 43%, and 31% respectively
Feng et al. (10)	N=14	Prospective	Stage I-III NSCLC	3DCRT	60 or 90 Gy	CT and PET-CT registration	After 40-50 Gy
Woodford et al. (11)	N=17	Retrospective	Stage IIIA (9), Stage IIIB (6), IV (2) NSCLC	Helical tomotherapy	60-64 Gy in 2 Gy per fraction	Merged MVCT-kvCT image sets	Daily
Duffton et al. (12)	N=12	Retrospective	12 NSCLC Stages T1-T4	3DCRT	55 Gy in 20 fractions	CBCT registration	Weekly
Wald et al. (13)	N=52	Retrospective	Locally advanced NSCLC	3DCRT/IMRT/VMAT	Mean 60 Gy, 2 Gy per fraction	kv-CBCT registration	Fraction 1, 11, 21, final
Yuan et al. (14)	N=56	Prospective	Stage I (N=11), II, (N-10), IIIA/B (N= 35) NSCLC	3DCRT	60 Gy	V/Q SPECT-CT scans and FDG-PET/CT studies	After 45 Gy
Moller et al. (15)	N=163	Retrospective	Stage T1-T4 (SCLC (N=46), NSCLC (N=117)	External beam radiation therapy	50–66 Gy in 2 Gy fractions for the NCSLC patients and 45 Gy in 30 fractions or 50 Gy in 25 fractions for the SCLC patients	CBCT registration	Daily
Zhang et al. (16)	N=34	Retrospective	NSCLC patients	–	Inst 1: mean 60 Gy, 2 Gy per fraction	CBCT registration	Weekly
					Inst 2: mean 66 Gy, mean 2.75 Gy per fraction		
Ramella et al. (17)	N=91	Prospective	Stage III NSCLC patients	Radiomics	–	–	–
Wang et al. (18)	N=9	Retrospective	LA-NSCLC patients	External beam radiation therapy	60 Gy	MRI registration	Weekly
Mehmood et al. (19)	N=27	Prospective	Stage II-III NSCLC	Chemoradiotherapy	60-74 Gy, 2 Gy per fraction	4D FDG-PET CT registration	Every 2 weeks
Yap et al. (20)	N=27	Prospective	Stage II-III LA-NSCLC	IMRT	60-74 Gy, 2 Gy per fraction	4DCT re-simulation	After Week 0, 2, 4
Agrawal et al. (21)	N=20	Prospective	Stage III LA-NSCLC	3DCRT	60 Gy	CT re-simulation	After 5 weeks
Xiao et al. (22)	N=17	Prospective	Stage II-III NSCLC	IMRT, 3DCRT	66 Gy	PET-CT re-simulation	After 40 Gy
Bertelsen et al. (23)	N=65	Retrospective	NSCLC patients	IMRT, VMAT	60/66 Gy, 2 Gy per fraction	CBCT registration	Every 10 fractions
Yartsev et al. (24)	N=17	Prospective	NSCLC patients	IGART	30 fractions	Merged MVCT-kVCT image sets	Daily
Van Timmeren et al. (25)	N=90	Retrospective	Stage II-IV NSCLC	Curatively intended radiotherapy	Mean 66.3 Gy	CBCT registration	Weekly
Chen et al. (26)	N=32	Retrospective	Stage II-IV NSCLC	IMRT, PSPT	60-74 Gy, 2 Gy per fraction	CBCT registration	Weekly
Berkovic et al. (27)	N=41	Prospective	Stage III NSCLC	IMRT	70 Gy, 2 Gy per fraction	kCBCT registration	Every fraction
Lim et al. (28)	N=60	Retrospective	LA-NSCLC patients	Radical RT	45 Gy or more	CBCT registration	Every fraction
30 Moller et al. (29)	N=63	Prospective	Stage I-IV NSCLC	IMRT	45/50/60/66 Gy	CBCT registration	Daily
Wang et al. (30)	N=8	Retrospective	NSCLC patients	IMPT	66 Gy, 2 Gy per fraction	4DCT registration	~34 days
Qin et al. (31)	N=6	Retrospective	NSCLC patients	VMAT	48-60 Gy	4D-CBCT registration	After treatment
Yang et al. (32)	N=38	Retrospective	Stage III NSCLC	PSPT (22), IMRT (16)	66/74 Gy, 2 Gy per fraction	4DCT re-simulation	Weekly
Spoelestra et al. (33)	N=24	Prospective	Stage II-IV NSCLC, SCLC	3DCRT	46 Gy	4DCT re-simulation	After 30 Gy
Finazzi et al. (34)	N=23	Prospective	Early stage NSCLC	SBRT	105/115.5/151.2 Gy	MRI registration	Every fraction
Henke et el (35).	N=12	Prospective	NSCLC patients	HSRT	60/62.5 Gy	MRI registration	Every fraction
Padgett et al. (36)	N=3	Case study	Stage IV NSCLC (N=1), Stage IB NSCLC, Stage IV pancreatic with metastasis to lung	SBRT (IMRT)	40-50 Gy	Deformable propagation to MRI	Daily

3DCRT, Three dimensional conformal radiation therapy; 3DCT, Three dimensional computed tomography; 4DCT, Four dimensional computed tomography; CBCT, Cone-beam computed tomography; CT, Computed tomography; HSRT, Hypofractionated stereotactic radiotherapy; IGART, Image-guided adapted radiation therapy; IMRT, Intensity modulated radiation therapy; kVCT, Kilovoltage computed tomography; LA-NSCLC, Locally advanced non-small cell lung cancer; MRI, Magnetic resonance imaging; MVCT, Megavoltage computed tomography; NSCLC, Non-small cell lung cancer; PET-CT, Positron emission tomography-Computed tomography; PSPT, Passive-scattering proton therapy; RBE, Relative biological effectiveness; RT, Radiotherapy; SBRT, Stereotactic body radiation therapy; SCLC, Small cell lung cancer; VMAT, Volumetric modulated arc therapy.

## Patient Selection for Adaptation Based on Changes in Tumor Volume

Currently, there is no uniform consensus regarding procedures to best select patients for adaptive planning. Several studies have identified using cone beam CT (CBCT) imaging to monitor tumor size, with potential shrinkage as a predictor of benefit from adaptive planning (9–11). Woodford et al. retrospectively investigated the use of daily Megavoltage (MV)-CBCT to track GTV changes in 17 patients with stage IIIA-IV lung cancer to determine a potential benefit of ART. The average change in tumor volume observed over 30 fractions ranged from -12% to -87% and could be broken into three groups with different advantages for adaptation based on the pattern of tumor volume change with time (11). Despite the limited sample size, the group proposed that a significant improvement in cumulative dose could be achieved by adapting plans where the volume tumor was reduced by 30% within the first 20 fractions.

Additionally, in a retrospective study, NSCLC patients [Stage II/III/-IV, mean 59.4 Gy three-dimensional conformal radiation therapy (3DCRT)/IMRT/volumetric modulated arc therapy (VMAT)] who underwent adaptive planning were determined to need replanning due to changes in tumor size and development of atelectasis. A reduction in GTV volume was observed in 35/58 patients (60.3%) and tumor shrinkage was found to be more probable in the middle and late phases of the treatment (p=0.049). These changes caused mediastinal shifts that would have led to poor coverage and additional toxicities if uncorrected (9). Adaptation for patients with mid and late phase reduction in GTV volume likely minimized local failure and adverse events.

In a prospective study, a subset of patients [Stage IIIA/B, 74 Gy passive scattering proton therapy (PSPT)] with plans adapted after 4DCT re-simulation in week 3 or 4 with large tumors (100 cm^3^) demonstrated reductions in esophagus V70 (mean absolute reduction 1.8%, range 0-22.9%, P< 0.01) and reductions in spinal cord maximal dose [median absolute change 3.7 Gy (RBE); range, 0–13.8 Gy (RBE)]. Increased tumor coverage was seen in 2/9 plans that would have had compromised CTV coverage without adaptive planning (6). In another study, Qin et al. retrospectively adapted 40 stereotactic body radiation therapy (SBRT) plans (Stage I and II, mean 49.25 Gy IMRT/3DCRT), in which 85% of the patients experienced a decrease in ITV volume, while the rest showed an increase throughout treatment. Adaptation was performed at each fraction of treatment and showed a significant reduction in OAR metrics for lung, esophagus, chest wall, and cord (P < 0.045), while experiencing large target size changes (mean change -21.0%, range -59.6-13.0%) throughout the course of the treatment (37). Adaptation may be of particular importance for SBRT patients with large target size changes to reduce dose to OARs. At this time, there is no consensus model for pre-determining changes in target size, although development of such a model would certainly facilitate patient selection for ART.

## Patient Selection for Adaptation Based on Physiologic Changes to OARs

Several studies have identified alternative factors that can be utilized to predict which patients benefit most from ART, including utilization of mid-treatment ventilation-SPECT (V-SPECT). Yuan et al. conducted a prospective study examining the effect of utilizing mid-treatment adapted ventilation/perfusion (V/Q) single photon emission computed tomography scan during RT in patients with Stage I-III NSCLC. Lung regions were classified into 5 categories consisting of: region A (tumor), region B1 (total functional loss), region B2, (reduced lung function), region B3 (temporary dysfunction lung because of tumor induced constriction) and region C (normal). Forty-three out of 56 patients (76.8%) with B3 regions had the improved V-SPECT, with 51.2% of those patients recovering completely or partially to normal function levels; 40/56 patients (71.4%) experienced improved perfusion, with 55% of those patients recovering completely or partially to normal function levels. These improvements in V/Q recovery may be linked to tumor shrinkage, caused by reducing pressure on the central airway and blood vessels, which can help identify patients who would benefit from ART (14). By identifying patients with tumor shrinkage using improvements in V/Q recovery, mid-treatment V-SPECT could be used as a trigger to determine patients who would benefit from ART.

Changes in lung volume due to atelectasis, pleural effusion, pneumonia/pneumonitis can also select for patients who may benefit from adaptive planning. While it has been shown that daily CBCT can reduce the risk of pneumonitis in unresectable NSCLC (38), ART may offer an even greater benefit. In a retrospective cohort of patients with lung cancer (Stage T1-T4, 46 SCLC and 117 NSCLC, 45-66 Gy 3DCRT/IMRT), 12% of patients who experienced atelectasis, pleural effusion, or pneumonia/pneumonitis experienced geometric shifts or dosimetric changes that would have benefited from adaptive planning, where atelectasis was the primary cause for adaptation. However, the results showed that decision to adapt the treatment plan cannot solely be based on the size and position of the atelectasis, but should be made on weekly evaluations through daily CBCT imaging (15). Although this strategy requires additional resources due to its reliance on weekly evaluations, it supports the inclusion of adverse events as a potential indicator of patients who benefit from ART.

Though most efforts around evaluating which patients would benefit most from ART have been related to tumor volume, there is a growing body of literature which also cites other indicators such as changes in V/Q scans and identification of pulmonary toxicities can aid in selecting patients for ART. Though analyses show that patients with shrinking tumors and pulmonary toxicity would benefit from ART, *a priori* prediction of changes in tumor size and toxicity due to radiation treatment would improve the clinical acceptance of ART.

## Predictive Models of Tumor Shrinkage, Toxicity, and Outcome

While patients with larger decreases in tumor volume have retrospectively been identified to benefit the most from ART (13), this fact can seldom be used in isolation to identify and select patients in advance for adaptive planning and treatment. There has been increasing effort placed in developing computational models to assist in assessing the need for adaptive radiation planning and treatment. Badawi et al. applied a principal component analysis (PCA) model to classify the geometric variability, which can come from daily setup variation, breathing, and treatment response of GTVs to guide adaptive therapy and improve treatment response. Twelve patients with locally advanced NSCLC undergoing curative radiation treatment underwent weekly breath-held CT. A physician contoured GTV was propagated using deformable registration to the subsequent weekly CTs; the Eigenmodes corresponding to tumor shape and position change and representing only time-dependent changes were used to create reduced models of tumor geometric variation. The prospective model, which utilized the data from the first 4 weeks of treatment was able to predict the positional variability (standard deviation of the GTV centroid) of future weeks with similar levels of error (1.8 ± 1.5 mm) to a retrospective model, which used all of the patient data (1.9 ± 1.4 mm) (39).

Tumor volume reduction in NSCLC patients treated with chemoradiation strongly correlates with post treatment survival (40). Ramella et al. developed a classification method (91 NSCLC, concurrent chemoradiation) based on clinical and radiomic features that was able to categorize tumors based on geometric differences and predict shrinkage due to radiation (AUC 0.82). Using machine learning to identify features that appeared in at least 10% of the iterations of the leave-one-out (LOO) loop, 5 semantic features (sex, tumor staging (N), histology, epidermal growth factor receptor (EGFR) mutation, and smoking attitude) and 6 radiomic image features were selected. The ability to predict the pattern of tumor shrinkage is a step towards helping patients achieve better tumor coverage and clinical outcomes (17).

Wang et al. retrospectively examined the use of a deep learning algorithm in ART to predict tumor regression. The P-net algorithm utilizes patches rather than whole images, which are identified as tumor or background based on the label of the center pixel. The features of each patch were extracted using a six-layer convolutional neural network (CNN) and fed into a recurrent neural network (RNN) that used those features to make a prediction. The predictions were compared to the actual tumor regression patterns. The validation accuracy of P-net was 95% and demonstrated the algorithm could predict spatial and temporal patterns of tumor regression using weekly MR imaging. Use of the algorithm on a single patient took 2 minutes, a significant improvement on manual adjustments, given the time consuming and costly nature of conventional ART workflow (18). Prospective studies of these algorithms are needed, but these results support that algorithms can quickly and accurately predict tumor regression patterns.

Instead of predicting changes in tumor size, other studies examined methods of predicting toxicities. In a prospective study, Mehmood et al. studied the correlation between mid-treatment FDG uptake and development of radiation esophagitis (RE) in patients (Stage II-III NSCLC, 60-74 Gy chemoradiotherapy). Patients underwent 4D-FDG positron emission tomography (PET)-CT scans every 2 weeks, which were co-registered to the planning scans for contouring and modified according to FDG uptake. A correlation was found between RE and an increase in the peak standard uptake value (SUVpeak) in weeks 4 (p=0.01) and 7 (p=0.03). Additionally, for grade 3 RE there was a strong correlation with the SUVpeak in week 2 (p=0.01) and 7 (p=0.03). Week 2 SUVpeak, may be a better predictor of development of grade 3 RE. Observation of these changes in FDG uptake can aid with selecting patients for ART by predicting patients who are more likely to develop RE (19).

Prior knowledge of tumor shrinkage and toxicities would improve the implementation and acceptance of ART by improving patient selection. Mathematical models can make such predictions based on indicators that have proven to be successful in estimating future outcomes without bias. The results of these studies show promise and the potential for future incorporation of ART. However, further modeling of the predictive benefits of ART with respect to changes in tumor size and motion is warranted. While knowing if a tumor will shrink or if a patient will have severe toxicities is important, pinpointing when the tumor will shrink and the most optimal time to adapt a plan is equally critical.

## Timing of Implementation of ART

In many clinical settings, the frequent scanning and replanning currently required to effectively use ART is not feasible. Additionally, even when plan adaptation might impact outcomes, it is not seen as an effective allocation of time or resources. To address this issue, studies have examined the ideal time to adapt treatments to benefit patients and improve the standardization and feasibility of ART. Studies have examined daily, weekly, and fractional intervals to assess the optimal time frame for treatment adaptation. In a prospective study, Yap et al. studied the feasibility of utilizing FDG-PET scans before and during chemoradiotherapy treatment to adapt treatment plans (Stage II-III NSCLC, 60-70 Gy IMRT). For each patient, 3 separate plans were created after treatment to study dose escalation based on PET scans at Week 0 (simulation/IMRT planning week), 2 and 4. Using a 50% SUVmax threshold, the dose in these areas was escalated to the maximum level that fell within OAR constraints for each plan. The results showed that the highest number of patients who were successfully boosted were from the Week 0 plan (n=24) compared to Week 2 (n=22) and Week 4 (n=19) (20). Although the plans were not applied prospectively and intermediate time points were not tested, the researchers concluded that the ideal time to utilize FDG-PET scans to escalate the dose in future prospective studies is at Week 0 because it allowed for the highest number of patients to be dose escalated.

Additionally, Agrawal et al. prospectively examined how tumor volume regression affected the treatment outcomes of a group of patients (Stage III NSCLC, 60 Gy 3DCRT). Each patient underwent a planning CT scan and another CT scan after 45 Gy. The initial treatment plan and a second plan (adapted based on both the initial CT and the CT after 45 Gy) were each evaluated by using a previously defined threshold of 60 Gy as the optimal dose for local control. The results showed that using only the planning CT, 30% of patients reached this threshold. However, using the additional CT after 45 Gy to adapt the plan, 80% of patients were able to have their dose escalated to 60 Gy. Despite the limitations posed by the small sample size, these results show potential for a replanning method at 45 Gy that does not require repeated imaging, which could be ideal for environments that lack resources (21).

Similarly, in a prospective study, Xiao et al. compared the dose volume histograms (DVHs) of an original plan to an adapted plan (17 Stage II-III NSCLC, 66 Gy IMRT/3DCRT). Each patient underwent FDG-PET scans once a week before treatment and another time after about 40 Gy. The group used metabolic tumor volumes (MTV) based on the FDG-PET as the GTV, with the original plan based on the pre-treatment MTV and an adapted plan based on the mid-treatment PET MTV. In order to evaluate the benefit of utilizing amid-treatment PET after about 40 Gy, the original plan was compared to a composite plan incorporating the adaptation and a statistical comparison of the differences in DVH values for the lung, heart, esophagus, and spinal cord was performed. The results showed improved DVH metrics for every OAR, including V5, V10, and V15 for lung (p<0.001), V10, V20, and V30 for the heart (p<0.002), V35, V40, and V50 for esophagus (p<0.001), and maximum dose for the spinal cord (p<0.004), among multiple others (22). The study demonstrated that mid-treatment PET scans can be used to define smaller target volumes, which improved overall dose to the OARs; however, this was a planning study and the efficacy of adaptation on tumor control was not assessed.

These studies provide a range of timings for adaptive planning that have shown the most promising results. Other studies generally support the 40-45 Gy range, as seen in studies that identify 20 fractions (11, 23, 24) or Week 3/4 (6, 25, 26) as an optimal time for adaptation. A few other studies identified either a slightly earlier range such as after 15 fractions (27, 28) or if feasible weekly scans (15). Overall, the results seem to favor adapting treatment slightly past midpoint or around the midpoint of treatment, as they attempt to wait long enough to detect changes in anatomy and dosimetry, but early enough to make an impact on efficacy, toxicity, and acknowledging the clinic workflow. Understanding when to adapt a patient’s plan is one critical way of improving clinical workflow for ART, minimizing the burden on the clinical schedule and staffing resources.

## Optimizing ART Workflow

In addition to finding the optimal timing, studies looked at other ways to combat the barriers posed by the additional costs and time associated with ART. To streamline the process of re-simulating and recontouring, some studies looked at ways to reduce time for certain steps or minimize the time physicians are needed. In a prospective study, Møller et al. examined the use of criteria based on daily CBCT scans to trigger a decision whether to adapt a patient’s plan (Stage I-IV NSCLC, 45-66 Gy IMRT). The criteria used included the tumor position, lymph node positions, thoracic vertebra positions, lung density changes, body contour changes, and mediastinum changes. Thirty-four percent of patients (79/233) were adapted and 75% of those adaptations (59/79) improved tumor coverage and reduced dose to healthy organs. The study was limited by an overall false positive rate of 20%, and these adaptations were deemed ineffective due to the nature of target shrinkage or the dose distribution counterbalancing the geometric shifts that triggered the adaptation. A slight change was made regarding the instructions given to the physicists to better correspond geometric change to realized dose consequences and only a 5% false positive rate was seen in the last 110 patients after the change (29). Using a set of objective trigger criteria rather than relying on a subjective decision maker allows for a more efficient workflow, reduction of interobserver bias, and simplification of the selection process for ART.

Other suggested methods to optimize workflow that appear in the literature include the use of neural network based algorithms (41), optimizing CT registration (30, 31), and maximizing the use of CBCT due to its short acquisition time (12). Reducing the additional costs and time of ART addresses one of its primary shortcomings and allows for more widespread use in different clinical settings.

## ART Across Different Radiation Techniques

Many studies investigate the use of different radiation techniques to optimize outcomes for patients who undergo adaptation. These studies attempt to identify whether a specific technique allows for more favorable results and can aid in the standardization of ART protocol. Yang et al. retrospectively evaluated the use of adaptive planning in patients (Stage I-IV NSCLC, 66/74 Gy IMRT/PSPT) who were treated with IMRT compared to those treated with PSPT with respect to locoregional failure. Given that proton therapy limits exit dose to healthy lung, it reduces radiation induced toxicity, at the cost of higher rates of local failure. Adaptive planning was performed if weekly scans showed a change in iGTV coverage of <95% target volume receiving less than 95% of prescribed dose or >2 cc receiving more than 120% of prescribed dose. Eighteen percent (38/212) of patient plans needed to be adapted, 12% of patients given IMRT had marginal failure (MF) compared to 9% of patients given PSPT, with similar rates of local failure (LF) and regional failure (RF) also observed (32). While adaptation was required 63% more often for PSPT patients, the results indicate that using PSPT with adaptive planning allows for greater protection of surrounding OARs without any significant differences in LF. Dose degradation due to the interplay effect, has limited the number of prospective studies regarding pencil beam proton scanning treatment techniques in lung cancer patients.

Likewise, Spoelstra et al. studied patients (Stage II-IV NSCLC/SCLC, 46 Gy) who underwent concomitant chemoradiotherapy and evaluated the tumor changes during a course of respiratory gated radiotherapy (RGRT). Using RGRT allows for smaller radiation fields which can reduce toxicities, but risks missing the target. As a result, significant changes in the size of the tumor can result in a loss of dose coverage to the tumor, resulting in a need for replanning. The results showed that only 1/24 (4%) of the patients met the criteria to undergo replanning (5% reduction in the PTV or excessive dose to critical organs due to tumor shrinkage) (33). As a result, ART proved to have a limited benefit for patients undergoing RGRT, compared to those undergoing more conformal radiation techniques. However, gated treatment techniques often lead to significantly longer treatment times, which in turn impact the clinical treatment schedule.

The data for PSPT showed promising results regarding LF patterns that warrant more prospective trials with analyses of survival; however, proton therapy is not widely accessible. While RGRT demonstrated less need for adaptation, the use of prospectively gated treatment techniques led to longer patient treatment times. While further research into the optimal radiation technique is needed, it should also be considered with respect to ideal daily imaging technologies and the clinical schedule.

## Use of MR-Based Treatment Technologies in ART

With key changes to treatment plans caused by tumor and organ movement, MRI-guided radiation therapy has emerged as a potential solution due to the ability to track tumor and organ movement with improved accuracy. In a prospective study, Finazzi et al. studied the use of stereotactic MR-guided ART in 23 consecutive lung cancer patients [4 NSCLC, 55-60 Gy, Stereotactic Ablative RT (SABR)]. Patients were evaluated at each fraction using 3D-MRI to determine whether their plans needed adaptation using factors such as tumor motion, previous lung radiotherapy, interstitial lung disease, or previous lung resection. Using this method of adaptation, they achieved BED_10Gy_ > 100 Gy in delivery of all fractions, which has been deemed a threshold for observing > 90% probability of tumor control. In addition to the improved tumor coverage, this procedure can reduce the adaptation process by about 10 minutes and does not require a radiation oncologist to be present for on-table QA as often (34). Although there were a few limitations such as the sample size and limited follow-up, the results showed reduced target volumes, successful delivery of all dosages, and more efficient workflow that warrants further study.

Additionally, Henke et al. retrospectively examined the effect of using MRI-guided ART on dosage to healthy tissue in a cohort of patients (NSCLC, 60 Gy HSRT). The patients received daily MRI imaging and were registered to the planning CT to create plans that were adapted mid-treatment. The results showed that at the mid-treatment point, 50% of patients had violations of the criteria used to determine feasible dose to OAR. After the mid-treatment adaptation, 100% of these patients had these violations corrected. In addition to the improvement of dose to OAR, the adaptive planning methods used in this study took on average 26 minutes (deemed clinically acceptable) and reduced necessary time because of the offline replanning (35). Despite the retrospective nature of the study, they were able to propose an efficient and effective adaptation method that they plan on testing prospectively.

Real-time adaptive therapy requires constant tumor monitoring during treatment. Dietz et al. used compressed sensing and principal component analysis in pursuit of real-time MR-guided ART. Six retrospective NSCLC data sets were used to demonstrate that acceleration factors of up to 10, could still produce images with low normalized mean square error while maintaining a Dice coefficient above 0.9 for tumor contours (42). However, further investigation is needed for the impact of directly under sampling clinical imaging data. In a separate study, Dietz et al. investigated convolution neural networks for real-time MR imaging reconstruction specifically aimed at adaptive therapy for MR-Linac hybrid systems. Free breathing balanced steady-state free precession images acquired on a 3T MR scanner from six NSCLC patients were retrospectively undersampled to simulate 5x and 10x acceleration. Tumor segmentation was performed by an auto-contouring program developed by the same group. Total CNN training took approximately six hours and produced image reconstruction times of 65 ms with mean Dice coefficients for tumor segmentation above 0.85 for all reconstructed data (43). However, the signal to noise ratio gain from using 3T MR images in conjunction with this image acquisition acceleration strategy, does not directly translate to the commercially available field strengths of MR-Linac hybrids (0.35T and 1.5T). Daily MR-IGRT represents state-of-the-art imaging in radiation therapy, however adapting treatment plans based on MR images rather than X-ray based technologies poses a myriad of QA questions.

## Use of Deformable Image Registration Based Algorithms in Place of Re-Simulation

Re-simulation for a mid-treatment patient, represents another significant burden on the clinic. For patients receiving ART, the process generally consists of utilizing repeat CT scans throughout the treatment for planning. Given the greater accessibility of CBCT scans in this process, researchers have focused on methods to utilize these scans to guide ART decisions with similar accuracy to decisions made by physicians (44). Studies such as Cole et al. (45) have identified deformable image registration (DIR) as more promising approach. Future research aims to develop DIR-based algorithms to register CBCT images to pretreatment CT scans to adjust treatment to tumor changes.

In a retrospective study, Yuan et al. examined the use of deformable registration to generate a virtual CT (vCT) to guide daily ART. Twelve patients (Stage III NSCLC, 60 Gy IMRT) underwent daily CBCT scans which were used to generate the vCT as well as a replanning CT (rCT) after 20 Gy. The vCT was retrospectively generated by deforming the planning CT (pCT) to the simulated CBCT using the Pinnacle Demons DIR algorithm (Philips Radiation Oncology Systems, Fitchburg, WI). By retrospectively comparing the vCT to the rCT collected during the study, they were able to examine whether using a vCT generated with the Demons DIR algorithm could accurately estimate the dose accumulation. The results showed a mean dose difference smaller than 1.5% for most metrics (PTV mean dose, lung CTV mean dose, esophagus mean dose, heart mean dose) but not spinal cord max dose. While the small sample size and CBCT image quality were identified as limitations, the results support the potential use of vCT in ART (46). The use of vCT instead of full re-simulation would minimize staffing and equipment needs, improve the turnaround time for offline adaptation, and minimize imaging dose to the patient.

Similarly, others have developed predictive models that utilize these DIR-based algorithms. A prospective study by Abdoli et al. examined the use of an average anatomy model (AAM) based on image registration (using the b-spline DIR technique) to reduce radiotherapy uncertainties caused by movement of the tumor. The AAM used registration of the CBCT to the pCT to estimate displacement of the tumor. While the registration was validated retrospectively, the AAM was validated prospectively on 15 patients (Stage II-IV NSCLC). The results showed a significant decrease in systematic errors using the rCT and AAM (up to 23% and 26% in vector length, respectively) compared to the pCT (47). The study was limited by the need to carefully select patients who would benefit from AAM as extreme deformations would lead to DIR inaccuracy. These techniques offer an improvement on use of a mid-treatment rCT while reducing the clinical workload and avoiding additional imaging time in the clinic and dose exposure from re-simulation.

While other studies have also identified pulmonary toxicities as predictors of DIR inaccuracy (48), the algorithms are continuously being improved to account for such changes and presence of similar conditions (49–52), and even moving towards accurately predicting tumor responses (53). While utilizing DIR algorithms with CBCT imaging is a promising solution to a full re-simulation, not all treatment modalities allow for daily CBCT imaging.

## Algorithms for Quality Assurance and Dose Reconstruction

There are several challenges with adaptive planning for patients with NSCLC including accurately deforming dose due to volumetric changes in the tumor, treatment quality monitoring, and real time treatment monitoring. Accurately estimating dose accumulated at the voxel level has significant consequences for quantitative assessment of tumor radiation response. ART is highly dependent on the DIR algorithm utilized for dose accumulation. In a retrospective study of commercial DIR software (b-spline based) and in-house built finite element method (FEM) algorithm on seven NSCLC patients with tumor regression, Zhong et al. demonstrated that the energy defect of deformable image registration map used for dose accumulation was a “useful tool” in assessment of accuracy of adaptive planning. The energy defect was significantly greater for the b-spline *versus* FEM based dose accumulations, 20.8 ± 13.4% *versus* 4.5 ± 2.4%, respectively (54). This metric proved useful in classifying uncertainty in deformable dose accumulation, which help with assessing accuracy of adaptive planning.

Zhong et al. used the principle of energy conservation, “radiation energy deposited on a geometric volume in an image must be equivalent to the radiation energy calculated from the reconstructed dose in the warped volume”, as a criterion for evaluating deformable dose mapping comparing a b-spine based commercial algorithm to an in-house built fast finite element method for dose mapping operations. Dose deformations from six NSCLC patients with tumor regression were used to demonstrate the improvement in dose mapping using energy conservation and the hybrid FEM technique with a mean error of 2.5 ± 1.9 mm (55). Currently, as there are no consensus guidelines to guide the QA process (3) of DIR dose accumulation, energy conservation looks like a promising tool to improve the QA process for ART.

Another major challenge for ART is effective QA. Zhong et al. developed a deformable lung phantom comprised of a motor-driven piston that compressed a heterogeneous sponge material with a tissue equivalent tumor composed of bolus in a sinusoidal pattern. 4DCT of the moving phantom was acquired and deformable image registration, using VelocityAI (Varian Medical Systems, Palo Alto, CA) and Elastix (University Medical Center Utrecht), was performed to calculate 3D dose at each phase using Pinnacle and EGSnrc. Thermoluminescent dosimeters (TLDs) were located within the simulated lung and tumor. TLD measurements were in good agreement for 3D dose calculated with Pinnacle using the Elastix DIR. However, the agreement in dose using the VelocityAI DIR was off by up to 11.8%. Agreement between the 3D dose calculated with EGSnrc using the Elastix DIR and the TLD measurements varied by up to 5.7% (56). In house QA solutions, represent a major step forward in improving the accuracy of ART, but the lack of commercialization or open-source development make these solutions inaccessible for most clinics.

Personnel requirements and inter-observer variability can make implementation of adaptive therapy burdensome in the clinic. In a retrospective study of six SBRT lung cancer patients, Qin et al, developed a clinical treatment dose reconstruction system based on CBCT imaging. The system integrated the treatment planning system (TPS), linac record and verify system, and CBCT imaging to semiautomatically monitor treatment dose. A novel phase-matching DIR system was developed, where the closest phase based on diaphragm position was identified from 4D-CT and 4D-CBCT, the images were then deformably registered to each other, the CT was cropped to match the field of view (FOV) of the CBCT, and CT number was then warped to the corresponding CBCT to generate a 4D-pseudoCT, which was utilized by the TPS for calculate doses on all phases (31). The results showed that the target registration error was analogous to inter-observer variability using publicly available lung landmark datasets, illustrating the effectiveness of this DIR-based dose reconstruction system. The effectiveness of the system on these patients demonstrates potential for future applications for patients who experience similar changes in tumor size. Additionally, the developed system could assist in routine treatment quality monitoring. Persoon et al. developed an in-house 3D portal dosimetry measurement system that when used in conjunction with kV-CBCT flagged patients with a gamma index > 1% in more than 5% of the CTV and changes in atelectasis > 7mm for re-simulation and re-planning (57). Automatically flagging patients for ART could assist in removing inter-observer bias in patient selection.

Mueller et al. developed a real-time markerless target tracking system for lung tumors for use with standard linear accelerators. An algorithm was developed to track the 3D target position during VMAT treatment delivery based on kV projections. Previous real-time limitations of the markerless target tracking were overcome using GPU-based computation. The CIRS lung phantom (Computerized Imaging Reference Systems, Inc., Norfolk, VA) and a modified HexaMotion platform (ScandiDos, Uppsala, Sweden) were used to simulate lung tumor motion accuracy of the tracking system. QA guidance was determined to ‘resemble’ TG-147 (58) and set up a framework for clinical implementation. Static localization accuracy, dynamic localization accuracy, and dynamic localization accuracy with treatment interruption passed QA requirements. The end-to-end latency of this technique was 230 ms. This study represents the first prospective implementation of markerless lung tracking (59). It also represents a big step forward in bringing real-time markerless image tracking to the clinic.

While the field is slowly starting to address the QA challenges of ART (treatment quality monitoring, automation, and real time tumor tracking for lung cancer patients) most of the solutions presented represent in-house products that are not commercially available. This shortfall greatly limits the access to improved and automated ART techniques for most clinics.

## Conclusion

Mid-treatment changes in tumor volume and toxicity continue to drive the need for ART in lung cancer patients. The clinical burden of ART is one of the major reasons it has not been widely accepted. However, the growing body of evidence shows overwhelming support for reduced toxicity while improving tumor dose coverage by adapting plans mid-treatment, and data appear favorable for tumor control. Predictive models of tumor shrinkage and toxicity, determining the optimum time to adapt a plan, creating clinically relevant optimal workflows, utilizing deformable image registration to minimize the need for re-simulation and improve the accuracy of dose accumulation, are just a few of the ways the field is moving toward a more uniform implementation of ART. However, further guidelines are needed to improve QA especially as real-time imaging and plan adaptation become more widely available. The use of targeted therapy and immune checkpoint inhibitors in combination with ART may offer improvements in outcomes while minimizing toxicities and warrants further study.

## Author Contributions

Study conception and design: SJ. Data collection: HP and DP. Analysis and interpretation of results: HP, DP, and MR. Draft manuscript preparation: HP, DP, SN, NY, SJ, and MR. All authors reviewed the results and approved the final version of the manuscript.

## Funding

Research reported in this publication was supported by the National Center for Advancing Translational Sciences (NCATS), a component of the National Institute of Health (NIH) under award number UL1TR003017.

## Conflict of Interest

The authors declare that the research was conducted in the absence of any commercial or financial relationships that could be construed as a potential conflict of interest.

## Publisher’s Note

All claims expressed in this article are solely those of the authors and do not necessarily represent those of their affiliated organizations, or those of the publisher, the editors and the reviewers. Any product that may be evaluated in this article, or claim that may be made by its manufacturer, is not guaranteed or endorsed by the publisher.
